# The first Jurassic coelacanth from Switzerland

**DOI:** 10.1186/s13358-022-00257-z

**Published:** 2022-09-22

**Authors:** Christophe Ferrante, Ursula Menkveld-Gfeller, Lionel Cavin

**Affiliations:** 1 Department of Earth Sciences, University of Geneva, Rue des Maraîchers 13, 1205 Geneva, Switzerland; 2 Natural History Museum Bern, Bernastrasse 15, 3005 Bern, Switzerland; 3 Department of Geology and Palaeontology, Natural History Museum of Geneva, CP 6434, 1211 Geneva 8, Geneva, Switzerland

**Keywords:** Sarcopterygii, Actinistia, *Libys*, New species, Mesozoic, Toarcian, Morphology

## Abstract

Coelacanths form a clade of sarcopterygian fish represented today by a single genus, *Latimeria*. The fossil record of the group, which dates back to the Early Devonian, is sparse. In Switzerland, only Triassic sites in the east and southeast of the country have yielded fossils of coelacanths. Here, we describe and study the very first coelacanth of the Jurassic period (Toarcian stage) from Switzerland. The unique specimen, represented by a sub-complete individual, possesses morphological characteristics allowing assignment to the genus *Libys* (e.g., sensory canals opening through a large groove crossed by pillars), a marine coelacanth previously known only in the Late Jurassic of Germany. Morphological characters are different enough from the type species, *Libys polypterus*, to erect a new species of *Libys* named *Libys callolepis* sp. nov. The presence of *Libys callolepis* sp. nov. in Lower Jurassic beds extends the stratigraphic range of the genus *Libys* by about 34 million years, but without increasing considerably its geographic distribution. Belonging to the modern family Latimeriidae, the occurrence of *Libys callolepis* sp. nov. heralds a long period, up to the present day, of coelacanth genera with very long stratigraphic range and reduced morphological disparity, which have earned them the nickname of ‘living fossils’.

## Introduction

Coelacanths, or Actinistia, is one of the two clades of living sarcopterygian fishes, the second one being the lungfishes, or Dipnoi. Only known by two extant species, coelacanths were more diverse in the past since their split from other sarcopterygians about 420 million years ago, although they were always very minority compared to other fish clades.

**Fig. 1:**
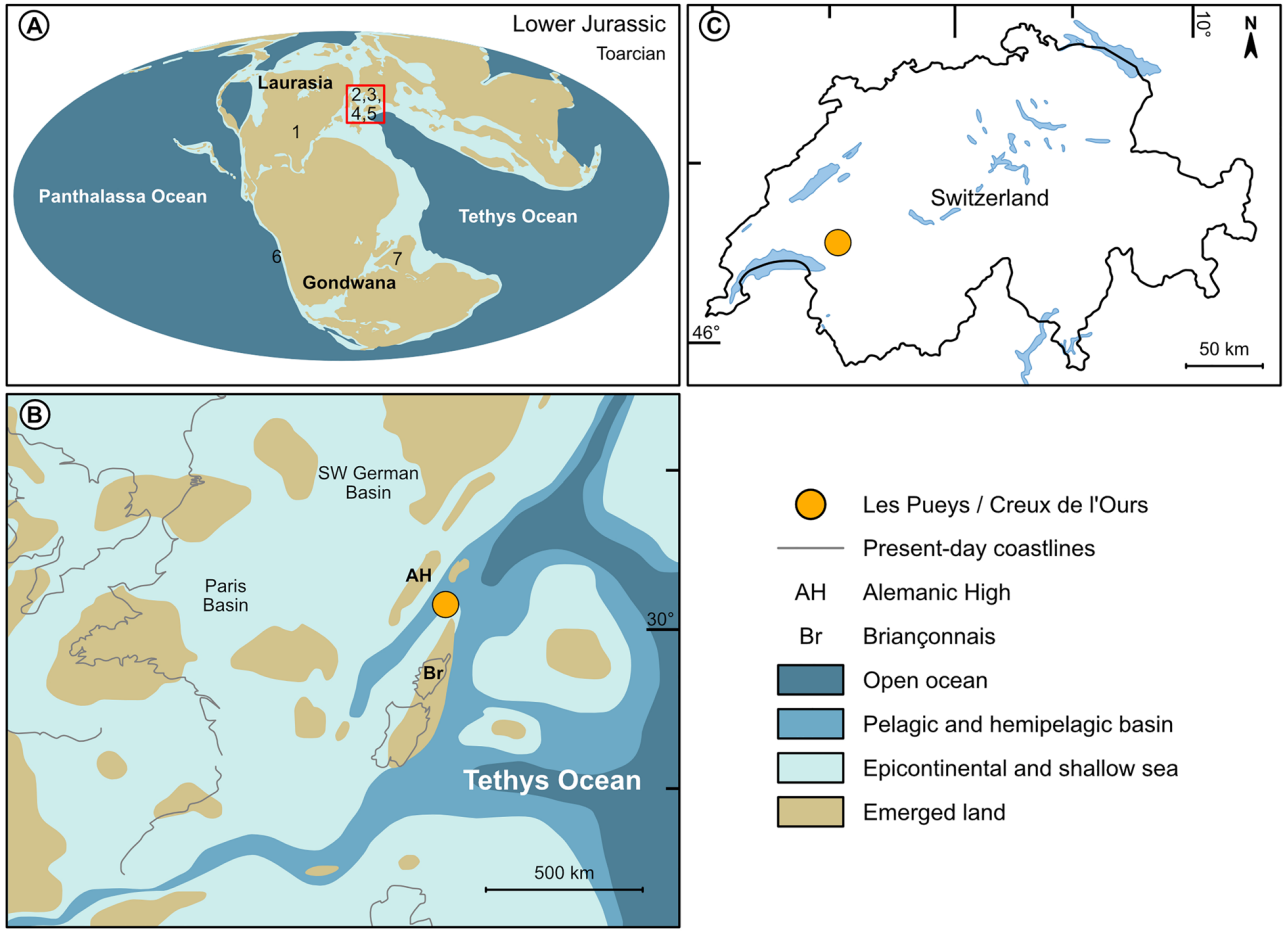
Maps showing the localization of the fossiliferous outcrops. **A** Paleogeographical map showing the continent configuration during the Toarcian (modified from Scotese, 2014, Map 39) with the distribution of (1) *Diplurus longicaudatus*, (2) *Holophagus gulo*, (3) *Trachymetopon*, (4) *Undina* (?) *barroviensis*, (5) *Libys callolepis* sp. nov. (6) *Atacamaia solitaria* and (7) *Indocoelacanthus robustus*. **B** Paleogeographic map of the NW Tethys Ocean (modified from Fantasia et al., 2019), enlargement of B (red square). **C** Map of Switzerland with the locality of Les Pueys near the Teysachaux summit (Canton of Fribourg, Switzerland)

In the Lower Jurassic, six species of coelacanths are currently known from marine and freshwater environments of Europe, North America, South America and India (Fig. 1A). Most species were recovered from different localities of Laurasia. The oldest known Jurassic coelacanth is *Diplurus longicaudatus* of the Hettangian-Sinemurian, a Laurasian species of which the genus is originated in the Carnian (Upper Triassic) and recovered from various freshwater sediments of United States (Fig. 1A1) (e.g., Forey, 1998; Schaeffer, 1948). Laurasian coelacanths are better known in marine environments of European localities. These coelacanths are represented by *Holophagus gulo* from the Sinemurian of England (Fig. 1A2), and the giant *Trachymetopon* from the Sinemurian and the Toarcian of Germany, the Callovian of France and the Kimmeridgian of England (Fig. 1A3) (Cavin et al., 2021a; Dutel et al., 2015; Forey, 1998). Another coelacanth of a poorly defined species of uncertain affinities, *Undina* (?) *barroviensis* has been described in the Lower Jurassic of England (Fig. 1A4) (Woodward, 1890, 1891; Forey, 1998). Gondwanian coelacanths are rarer during the Lower Jurassic and are represented only by two known species. The marine *Atacamaia solitaria* is the only known coelacanth that lived on the Paleopacific side of Gondwana (Fig. 1A6) during the Middle to Upper Sinemurian (Arratia & Schultze, 2015). *Indocoelacanthus robustus*, recovered from the Kota Formation in India (Fig. 1A7), is the only known coelacanth that lived in the freshwater environments of Gondwana during the Toarcian (Jain, 1974). This low taxic diversity of coelacanths during the Lower Jurassic makes a new occurrence of coelacanth particularly interesting for understanding the evolutionary history of the group.

In Switzerland, coelacanths are well represented in the Middle Triassic with *Ticinepomis peyeri* (Rieppel, 1980), *Heptanema paradoxum* (Renesto & Stockar, 2018; Renesto et al., 2021) and a new taxon currently under description (Ferrante et al., 2017; Rieppel, 1985; Ferrante et al., work in progress), all recovered from the marine environment of Monte San Giorgio and Ducanfurgga (Canton of Ticino and Graubünden, respectively). We report here the presence of a specimen of a coelacanth (NMBE 5034072 and 5034073) from the Toarcian, Early Jurassic, of the Swiss Prealps of the Canton of Fribourg. This specimen is then the very first coelacanth in Switzerland from another period than the Triassic. Moreover, this specimen collected in 1870 at the locality of Les Pueys (Fig. 1C) near Teysachaux (Canton of Fribourg, Switzerland), represents probably the very first coelacanth fossil found in Switzerland. The identity of the collector is not assured. It was probably Joseph Cardinaux, from Châtel-Saint-Denis (Canton of Fribourg, Switzerland), a fossil collector and dealer. Joseph Cardinaux found and sold many other fossils, notably the first ichthyosaur from Switzerland (Mennecart & Havran, 2013), also from Teysachaux, purchased by Carl von Fischer-Ooster, a paleontologist at the Naturhistorisches Museum Bern (Canton of Bern, Switzerland). When Carl von Fischer-Ooster incorporated the coelacanth specimen in the collections in 1873–1874, he gave it the name *‘Macropoma heeri’*, but never properly describing it, probably because he passed away in 1875. This name is then a *nomen nudum* according to the International Code of Zoological Nomenclature. Since its discovery, the specimen has only been mentioned as ‘*Macropoma*’ by Hug (1898) in its description of the fauna of Les Pueys and by Marchant and Pichon (2013) in their general guide summarizing the essentials of paleontological discoveries in Switzerland.

## Geological and paleogeographic settings of the Teysachaux sites

The coelacanth specimen described here was recovered from a small locality named Les Pueys near the village of Châtel-Saint-Denis (Canton Fribourg, Switzerland) (Fig. 1C). This locality is situated on the western slope of the Teysachaux summit, which is on the same mountain ridge than the emblematic Swiss Moléson summit. The Teysachaux region is best known for another fossiliferous site named Creux de l’Ours (Fig. 1C) which was exploited since the nineteenth century. The site of the Creux de l’Ours has yielded many marine fossils, including bivalves, gastropods, ammonites, belemnites, decapods, echinids, trace fossils, plant remains and some rare vertebrates such as fishes and one almost complete skeleton and some remains of ichthyosaurs (Fischer-Ooster & Ooster, 1870; Furrer, 1960; Huene, 1939; Menkveld-Gfeller, 1998; Mennecart & Havran, 2013; Weidmann, 1981). In the sites of Creux de l’Ours and Les Pueys, first fossils were discovered and collected mainly by the amateur collector Joseph Cardinaux, who sold many of his finds to the Naturhistorisches Museum Bern (Canton of Bern, Switzerland). From the Creux de l'Ours site, more than 400 fossils and from the site of Les Pueys about a hundred fossils were sold to the Naturhistorisches Museum Bern. Fischer-Ooster and Ooster (1870), Hug (1898), Huene (1939) and von der Weid (1960) described the fauna of these two sites in detail. The name of the collector who found the coelacanth of Teysachaux is unknown, but according to the dates of collect (before 1870) and of arriving in collection of the Naturhistorisches Museum Bern (1873–74), it is likely that it was Joseph Cardinaux.

**Fig. 2:**
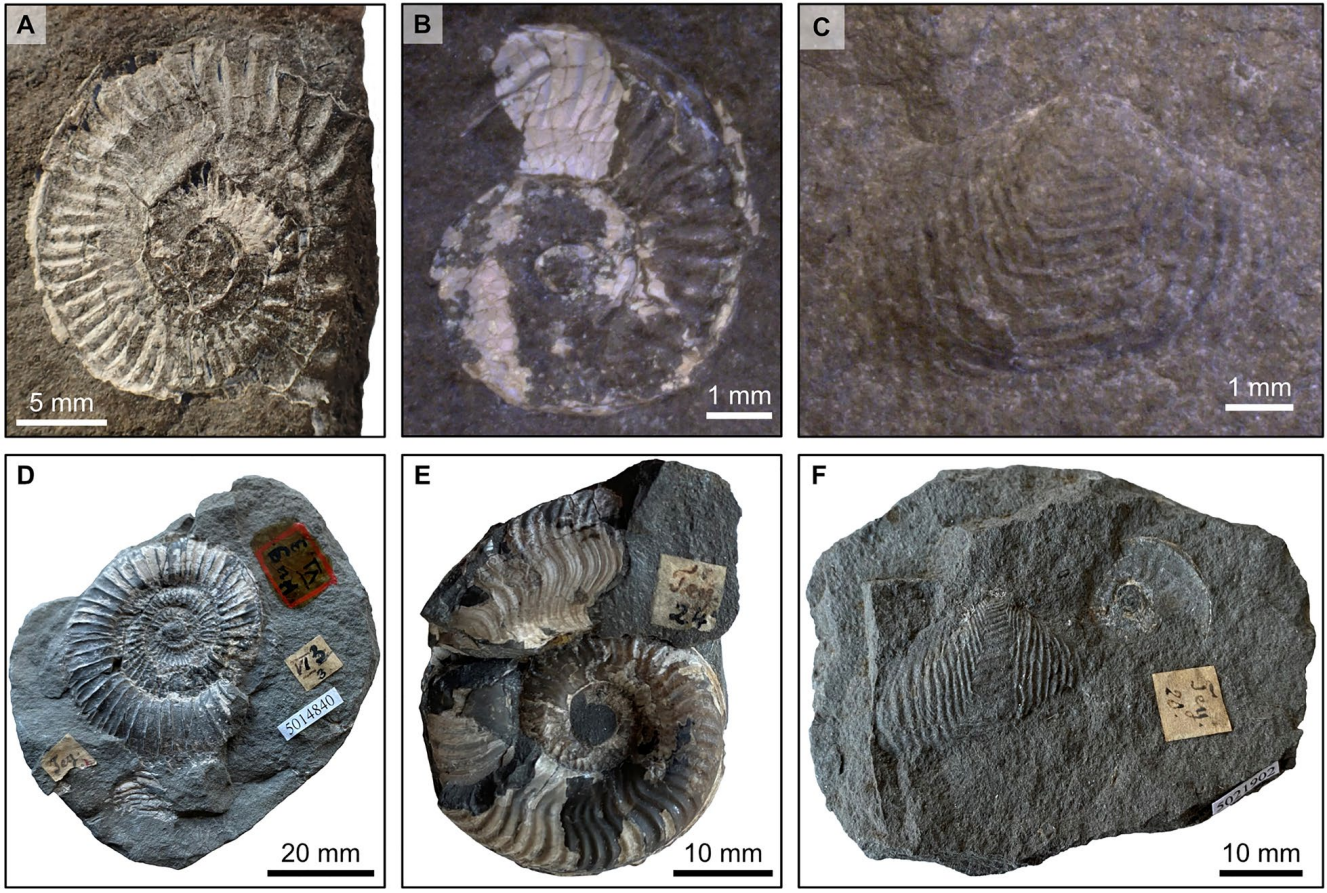
Ammonites and bivalves of Les Pueys compared to ammonites and bivalves of the Creux de l’Ours. Invertebrate fossils **A**–**C** preserved on the reverse side of the two slabs with *Libys callolepis* sp. nov. (holotype, NMBE 5034072 and 5034073) from Les Pueys and **D**–**F** invertebrate fossils from the Creux de l’Ours. **A** *Dactylioceras*? sp. (NMBE 5034073); **B** *Harpoceras renevieri* (NMBE 5034072); **C** *Goniomya rhombifera* (NMBE 5034072); **D** *Dactylioceras commune* Sowerby 1815 (NMBE 5014840); **E** *Harpoceras renevieri* (holotype, NMBE 5014830); **F** *Goniomya rhombifera* (NMBE 5021902)

The exact location from which the coelacanth material was recovered is not clear but Hug (1898) mentioned that the fossil beds of Les Pueys are found in a small river. Prospecting near the place called Les Pueys carried out by one of us (CF) permitted to find a small valley crossed by a small river with some fossiliferous layers that could correspond to the site mentioned by Hug (1898). Comparison of the lithology and the invertebrate fossils on the slab containing the coelacanth specimen with the material from the Creux de l’Ours bonebeds suggests that the coelacanth material of Les Pueys is part of the same lithostratigraphy than the Creux de l’Ours site. Indeed, on the reverse side of the two slabs with the coelacanth (NMBE 5034072 and 5034073) are various small coprolites, fish scales and fine fragments of bones, shell fragments and some poorly preserved evolute forms of ammonites (Fig. 2A, B). The latter are referred to *Dactylioceras*? (Fig. 2A) and *Harpoceras renevieri* Hug 1898 (Fig. 2B) of the families Dactylioceratidae and Hildoceratidae, respectively. A bivalve was identified as *Goniomya rhombifera* Goldfuss 1840 (Fig. 2C). Those fossils also resemble to the findings from the same strata of Creux de l'Ours in terms of the manner in which the shell is preserved. The same invertebrate genera and species are also known from the Creux de l'Ours site (Fig. 2D–F).

The Creux de l’Ours section is part of the Stadelgraben Formation from the Nappe of the Préalpes Médianes Plastiques (e.g., Fantasia et al., 2018; Weidmann, 1993). Septfontaine (1983) introduced the term Staldengraben Formation for this assemblage of beds, subdivided in ‘units’ A, B, C and D. Weidmann (1993) then assigned the fossil sites to the Staldengraben Formation, more precisely to the Unit A. The exceptional character of these beds (i.e. good preservation and abundance of fossils and organic matter content) seems to be a consequence of the Toarcian oceanic anoxic event (Mettraux, 1988; Mettraux & Mosar, 1989). Plancherel et al. (2020) later proposed to use the term Staldengraben Formation, but to redefine the Members by adding names of localities where the corresponding lithologies are particularly well represented (proposed type localities). Thus, the layer, from which the coelacanth was collected, is assigned to the Soladier Member of the Staldengraben Formation. The Soladier Member is composed by sediments predominantly clayey with alternation of dark schistose marls, altered to brown-beige, and light marly limestones, mottled, in thin banks (10–30 cm). The coelacanth fossil lies on the cleavage surface of a light-grey to brownish marly limestone that is then characteristic from the Soladier Member.

The sediments of the Creux de l’Ours section (Fantasia et al., 2019) and of Les Pueys were deposited in the western Tethys in a deep and distal part of the Sub-Briançonnais basin bordered to the south by the Briançonnais micro-continent and to the north by the Alemanic High (Fig. 1B). Hug (1898) dated the Creux de l’Ours beds and of Les Pueys to the early Toarcian based on ammonites. Weidmann (1993) noted that recent collections of ammonites, carefully collected bed by bed, have revealed the presence of the *Elegantulum* and *Exaratum* Subzones of the Falcifer Zone (Pugin, 1985) dated of the Toarcian. During the Toarcian, the marine environment has experienced an important anoxic event known worldwide as the ‘Toarcian oceanic anoxic event’, which is marked by marine mass extinctions with a global warming, a perturbation of the carbon cycle and important depositions of organic-rich sediments (Fantasia et al., 2018). Sedimentological analyses in the Creux de l’Ours section show high kaolinite contents and detrital proxies suggesting warm and humid climate during the ‘Toarcian oceanic anoxic event’ (Fantasia et al., 2018). The sediments of the Creux de l’Ours section (Fantasia et al., 2018) and of Les Pueys are composed of grey thin-bedded hemipelagic marls and marly limestones with carbonate concretions formed around accumulations of ostracods and gastropods shells.

## Systematic paleontology

**Sarcopterygii** Romer 1955

**Actinistia** Cope 1871

**Latimerioidei** sensu Toriño et al., 2021

**Latimeriidae** sensu Toriño et al., 2021

***Libys*** Münster 1842

**Diagnosis** (emended from Forey, 1998)

A genus of medium-sized, relatively deep-bodied coelacanths. The head is nearly as deep as long. The postparietal shield is much expanded posterolaterally where the supratemporals are particularly large. The palate is deep and tapers rapidly anteriorly and the symplectic is also long, in keeping with the rather deep head. The cheek is covered with large thin bones which are, however, well separated from one another. The postorbital and lachrymojugal are broad and the preopercle is long and strap-like. The opercle is substantially deeper than broad. The subopercle and the preorbital are absent. Sclerotic ossicles are present. In the lower jaw the angular shows a prominent dorsal expansion and the principal coronoid is developed posterodorsally as a rounded finger-like process. The most obvious specialization of *Libys* are the sensory canals which open to the surface through a large groove crossed by pillars. Ornamentation is absent from the skull bones. The shoulder girdle shows a very narrow cleithrum, clavicle and extracleithrum. The anocleithrum is simple and sigmoid to blade-like. The pelvic fin is located well behind the level of the anterior dorsal fin and is supported by very narrow pelvic bones. The rays of the anterior dorsal fin and the caudal fin are ornamented with many prominent denticles. Fin rays of pectoral, ventral, posterior dorsal and anal fins are very closely articulated close to their bases. The supplementary caudal fin is prominent developing apart from the caudal fin profile. The lateral line scales carry a large sensory tube which opens through several secondary tubules. An ossified lung is present.

***L. polypterus*** Münster 1842

**Diagnosis**

*Libys* species with the postparietal shield less than half the length of the parietonasal shield. The teeth covering the palate and the lower jaw are very small, and most are rounded and bear delicate radiating ridges. 70 neural arches. Fin rays of the posterior dorsal, pectoral, pelvic and anal fins are expanded. The pectoral fin is relatively long, reaching back to posterior level of pelvic fin. The scales are covered with a sparse ornament of short ridges.

**Measurements and meristic**

(SL) Standard length 600 mm.

d1.f = 10; d2.f = 15–20; pect.f = 16; pelv.f = 19; ana.f = 18–20; cau.f = 21/19; n.a = 70; h.a = 23.

**Holotype**

BSM 1870.xrv.502, head only.

**Horizon and type locality**

Tithonian (Upper Jurassic), Bavaria, Germany.

***L. callolepis*** **sp. nov**.

**Diagnosis**

*Libys* species with the postparietal shield about half the length of the parietonasal shield (the parietonasal is then proportionally shorter than in the type species). The teeth covering the prearticular are very small, and rounded and smooth. Between 41–47 neural arches. Fin rays are slender than in the type species and then not expanded. The scales are strongly ornamented with irregularly sized and elongated round-to-ovoid ridges disposed along a longitudinal axis.

**Measurements and meristic**

(TB) Total body length 290 mm (estimation); (SL) standard length 255 mm.

d1.f = 10; d2.f = 16; pect.f = 18–22; pelv.f ≥ 17; ana.f = 20–23; cau.f = 15/14–16; n.a = 44–47; h.a = 18–20.

**Etymology**

From the ancient Greek καλός, kalós, (‘beautiful’, ‘nice’) and λεπίς, lepís, (‘scale’) in reference to the nicely ornamented scales of the species, which differentiates it from the type species.

**Holotype and only known specimen**

NMBE 5034072 and 5034073, a sub-complete specimen preserved in right lateral view as part and counterpart. Most of the bones, including the scales on the body, are preserved in anatomical position and only the bones of the cheek and the jaw are missing. The specimen is kept in the collections of the Natural History Museum Bern (Canton of Bern, Switzerland).

**Horizon and type locality**

Toarcian (Lower Jurassic), Creux de l’Ours section, locality of Les Pueys near the Teysachaux summit (Canton of Fribourg, Switzerland).

**Nomenclatural act**

The present work and its nomenclatural act are registered in ZooBank, the online registration system for the International Commission on Zoological Nomenclature. The Life Science Identifiers for this publication is “urn:lsid:zoobank.org:pub:D03A2AC9-51F9-45E6-8CAC-F06A1526AF2C” and can be resolved appending the prefix “http://zoobank.org/” in any standard web browser.

## Description

### Generalities

**Fig. 3:**
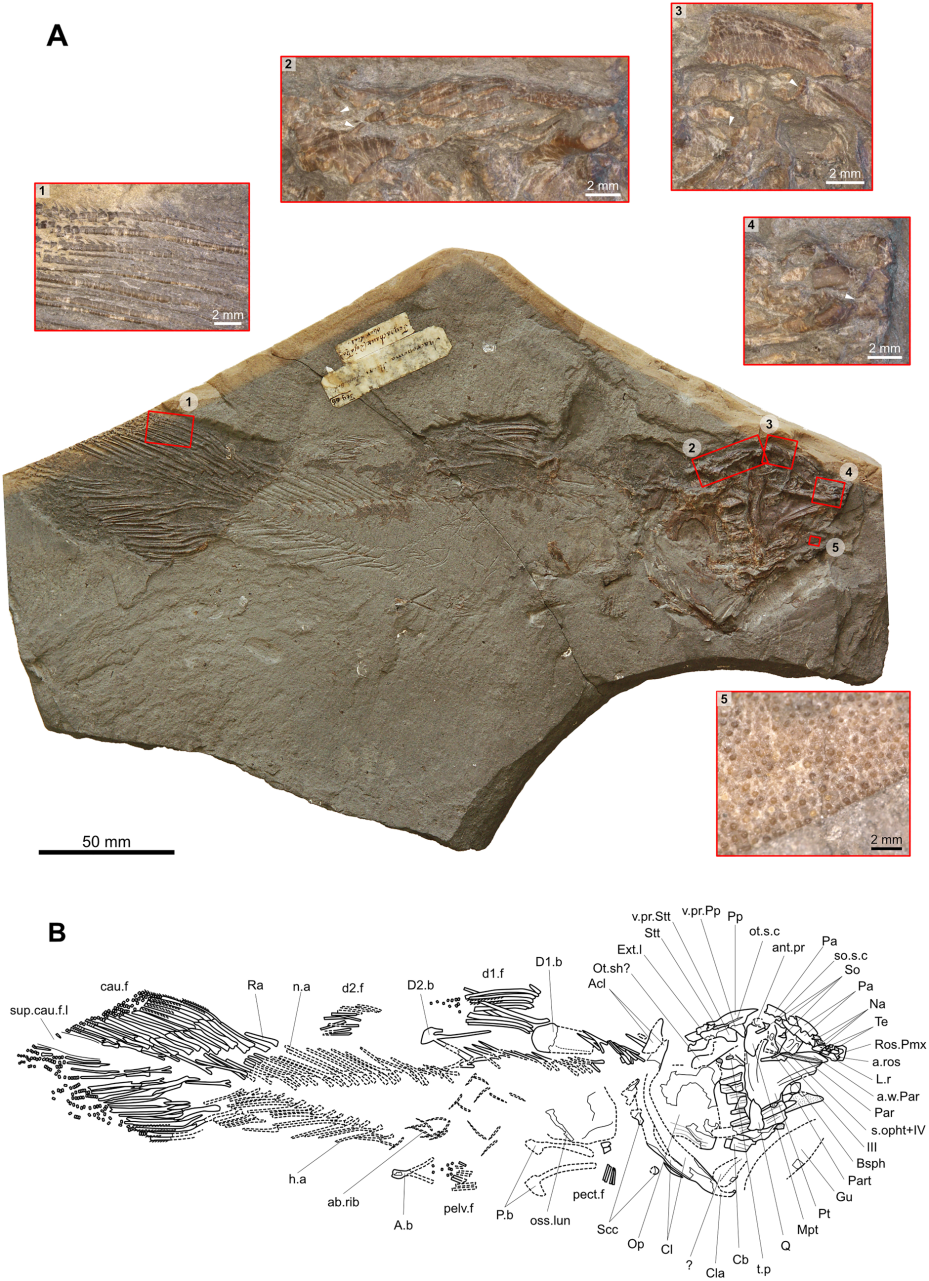
Skeleton of *Libys callolepis* sp. nov. on the part (holotype, NMBE 5034073). **A** Photos with osteological details: 1, denticles on the proximal fin rays of the caudal fin. 2, Postparietal shield with the otic sensory canal opening as a deep groove crossed by pillars (white arrowhead). 3, Posterior parietal and the supraorbitals with their pillars (white arrowhead). 4, Consolidated snout with the anterior opening for the rostral organ (white arrowhead). 5, Teeth on the prearticular. **B** Semi-interpretative line drawing of the specimen

**Fig. 4:**
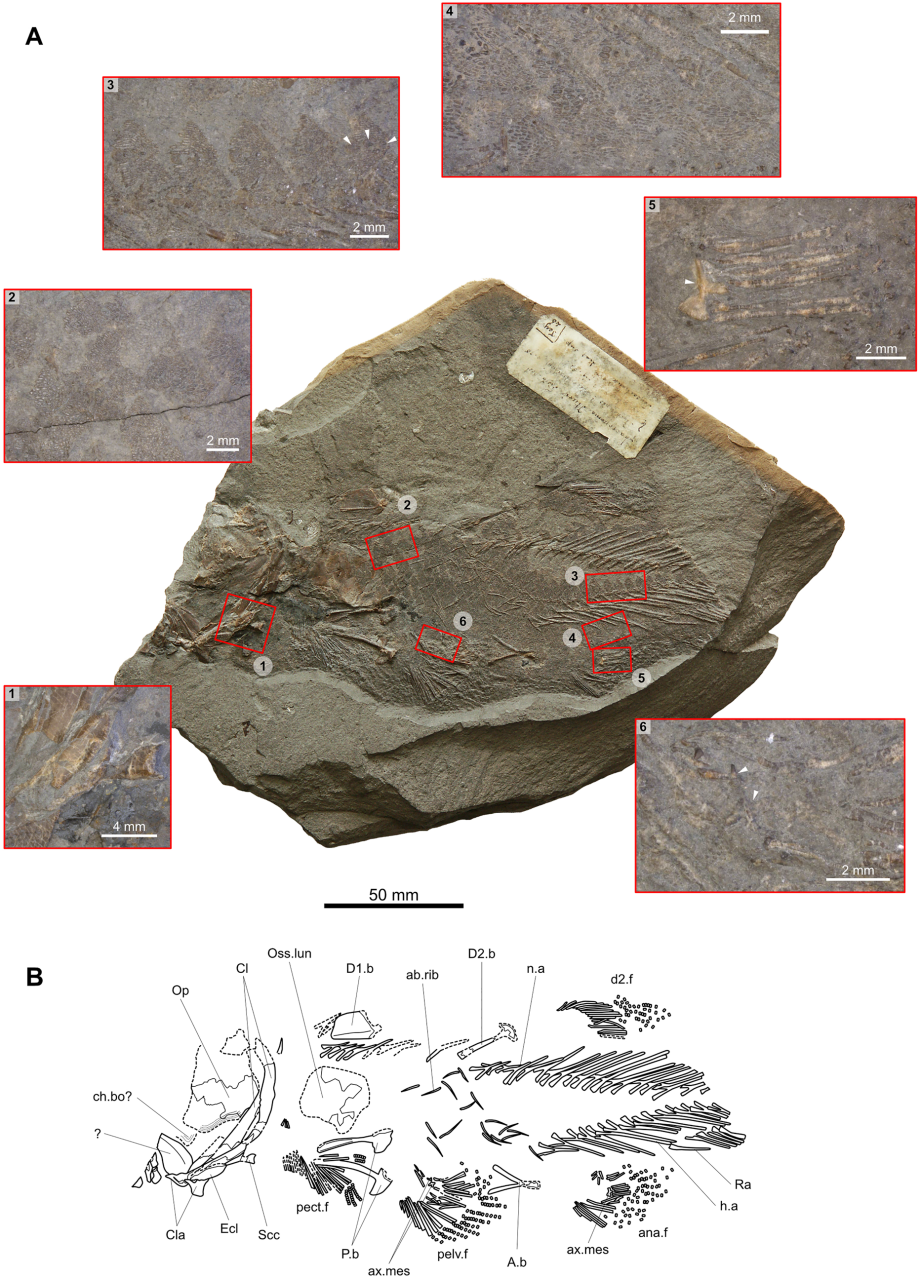
Skeleton of *Libys callolepis* sp. nov. on the counterpart (holotype, NMBE 5034072). **A** Photos with osteological details: 1, articular head of the scapulocoracoid. 2, Scales on the flank immediately beneath the first anterior dorsal fin. 3, Scales of the lateral line showing the ornamental pattern with the larger central tubercles (white arrowheads point, showed only on one scale). 4, Scales on the ventral flank from the pelvic to the anal fin. 5, Axial mesomere (white arrowhead) surrounded by some fin rays of the anal fin. 6, Axial mesomeres (white arrowhead) partially covered by sediment in the pelvic fin. **B** Semi-interpretative line drawing of the specimen

Most of the bones of the specimen are preserved on the part (NMBE 5034073) (Fig. 3). From the head, most of the dermal bones of the skull roof are preserved. The bones of the cheek and the lower jaw are missing, revealing then the bones of the palatoquadrate and of the branchial apparatus. The axial skeleton, the girdles and the fins are almost entirely preserved, except the posterior tip of the supplementary caudal fin lobe. The scales are well preserved on the entire body in natural position, especially on the counterpart (NMBE 5034072) (Fig. 4).

The specimen may represent an adult individual as all the basal plates are fully ossified, which is a feature observed in adult coelacanths (e.g., Schultze, 1980; Witzmann et al., 2010). This view is reinforced because the specimen shows some well ossified axial mesomere and the scapulocoracoid (Fig. 4A1, A5, A6, B). Although a long supplementary lobe of the caudal fin is considered as a juvenile character (e.g., Forey, 1981; Schultze, 1972), the prominent supplementary lobe of the caudal fin (Fig. 3) observed in the specimen of Teysachaux represents rather a generic character of the specimen.

### Dermal bones of the skull roof

**Fig. 5:**
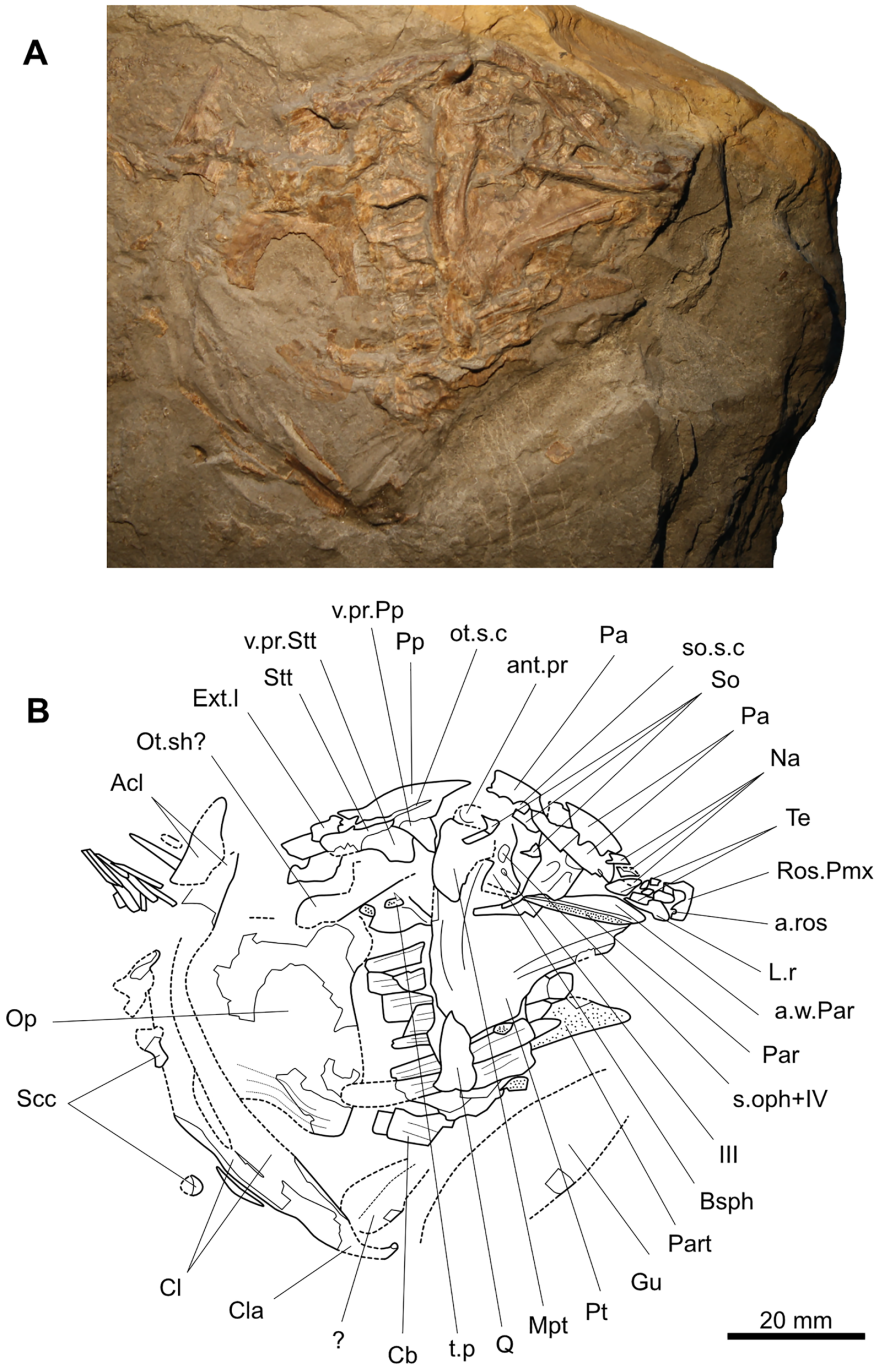
Skull of *Libys callolepis* sp. nov. on the part (holotype, NMBE 5034073). **A** Photos and **B** outline of the skull

The bones of the skull are only preserved on the part (Figs. 3 and 5). The skull roof is divided into a parietonasal and postparietal shields, free from one to the other and separated by the intracranial joint, which appears to be straight. The postparietal shield appears to be smaller than the parietonasal shield, this last being about 1.45 longer. Although slightly lower, this ratio is however close to the ratios of Jurassic and Cretaceous coelacanths that have typically a parietonasal shield circa 1.5 to 2 times longer than the postparietal shield (e.g., *Libys polypterus* has a ratio of about 1.7). On the Teysachaux specimen, the dermal bones of the skull appear to be smooth and unornamented.

#### Parietonasal shield

The tip of the snout (Fig. 3A4, B and Fig. 5) is developed as a heavily ossified hemisphere (Ros.Pmx), which it is not sutured to the neighboring lateral rostral and rostral ossicles or nasals. It is difficult to discern the premaxilla in this structure, but the presence of this bone can be deduced because of a posterior notch that is interpreted here as the anterior opening for the rostral organ (a.ros). No teeth are observed, but this could be due to taphonomic processes. This consolidated snout is reminiscent of the ossified hemispherical snout of *Macropoma lewesiensis* (Forey, 1998, f﻿igs. 3.19 and 3.20). The difference is that this consolidated snout presents no ornaments, while *M. lewesiensis* has a consolidated snout strongly ornamented with small rounded tubercles (Forey, 1998).

An elongated bone posterior and ventral to the consolidated snout is interpreted as a lateral rostral (L.r) (Figs. 3 and 5). This bone has been compressed inside the skull making the ventral process to point laterally.

Posterior to the consolidated snout lie at least three nasals (Na) (Figs. 3 and 5). The nasal in the middle of the series is crushed and broken. It is unclear if the sheets of bone that lie between the anterior nasal and the consolidated snout are small rostral ossicles, or a fragmented nasal. The posterior two nasals are of the same width than the parietals, and only the anterior most nasal is slightly narrower. It is difficult to assess the length of the nasals because of their preservation, but it seems that they are all of the same length and considerably shorter than the parietals. The lateral margin of the posterior most nasal has elongated bony pillars that separate large openings of the supraorbital sensory canal (so.s.c).

The right anterior parietal (Pa) is followed posteriorly by the right posterior parietal (Figs. 3 and 5). The right anterior parietal has its posterior margin crushed. The anterior portion of the posterior parietal is covered by sediments and crushed bone, making difficult to assess its length. However, regarding the length of the anterior parietal, it is likely that both parietals have the same length. Below both parietals is a long bone of the left side preserved in internal view. It is unclear if this bone represents a single bone (a parietal) with a fracture in its middle or two bones (two parietals or one parietal plus a nasal). Regarding the present elements, it appears that both parietals have almost the same width. Unfortunately, it is unclear if the descending process of the parietal is present or not. The lateral margins of the parietals produce thin bony pillars, which are much more elongated on the anterior parietal (Figs. 3A3, B and 5). These pillars, when in contact with the neighboring supraorbitals, delimitate very large openings of the supraorbital sensory canal (so.s.c).

From the lateral series, four posterior supraorbitals (So) can be clearly observed near the lateral margin of the posterior parietal (Figs. 3 and 5). Compared to the length of the parietals, it is clear that there were more supraorbitals composing the lateral series. Only one supraorbital lies directly in contact with the posterior parietal while one or two posterior most elements are not in direct contact. The supraorbitals have their mesial margin producing bony bars or pillars that directly contacts their antimeres on the parietals (Figs. 3A3 and B and 5). The openings of the supraorbital sensory canal are then proportionally large, almost forming a groove crossed by pillars. A supraorbital sensory canal opening through a continuous groove crossed by pillars is reminiscent of *Libys polypterus* and *Megalocoelacanthus dobiei* (Dutel et al., 2012). However, compared to the two latter taxa, the groove/openings in the specimen of Teysachaux is a bit less wide. The lateral bony portion of the supraorbitals are narrower compared to the parietals, but with the pillars the entire bone is almost as large as the parietals. Regarding the way that the sensory canal opens, it probably follows a sutural course between the parietals and the supraorbitals as in most coelacanths.

Apart from these four supraorbitals, two elements representing tectals (Te) can be observed in the snout (Figs. 3 and 5). These small bony plates are of the same width than the supraorbitals. Due to the poor preservation of the lateral series, it is then impossible to determine the exact number of elements within the series with accuracy.

No preorbital is preserved on the specimen and its presence/absence remains unknown.

#### Postparietal shield

The postparietal (Pp) appears to be of the same width than the posterior parietal and supraorbitals together (Figs. 3 and 5). Below the right postparietal extends a large ventral process (v.pr.Pp). Unfortunately, the state of preservation makes impossible to observe if anterior branches of the supratemporal commissure, the median branch of the otic canal and pit lines of the postparietals are marking the bone.

The supratemporal (Stt) is half as long as the postparietal (Figs. 3 and 5). The supratemporal bears a large ventral process (v.pr.Stt) stouter than the ventral process of the postparietal. The otic sensory canal (ot.s.c) separates the postparietal from the supratemporal as a deep groove but meet together by thin bony pillars lying within the groove (Figs. 3A2, B and 5).

The supratemporal exceeds the posterior limit of the postparietal and enclosed a badly crushed bone that is interpreted here as a lateral extrascapular (Ext.l) (Figs. 3 and 5). Posterior to this bone lies a bone visible in internal view that could correspond to another extrascapular. The state of preservation makes difficult to assess if the extrascapulars are sutured or are free from the postparietals. The size of the extrascapular suggests that there were few bones in the extrascapular series, which is a condition similar to *Libys polypterus* and unlike *Macropoma* (Forey, 1998). The extrascapular being situated behind the level of the neurocranium indicates that the braincase has not fused with the extrascapulars.

All the dermal bones of the skull roof are completely smooth. The situation is then similar to *Libys polypterus*, to the contrary of *Macropoma* that has the dermal bones of the skull covered with small round tubercles (Forey, 1998). The parietals and postparietal bear no raised areas.

### Dermal bones of the cheek

No cheek bones are preserved. Only a very badly preserved opercle (Op) is present (Figs. 3–5). Although the general shape is difficult to assess, the imprint and the remaining bone show that the opercle is pinched-out ventrally as in other actinistians as for instance in *Libys polypterus* or *Macropoma lewesiensis*. The preserved portions of the opercle on the part present no ornamentation and are smooth. On the counterpart, anteriorly and below the opercle is a poorly preserved imprint of small scale-like bone with lines of growth, which may possibly correspond to a bone of the cheek (ch.bo?), such as for instance the preopercle or subopercle (Fig. 4).

No sclerotic ossicles are observed in the specimen of Teysachaux but the state of preservation precludes to assess a true absence. In his emended diagnosis of *Libys*, Forey (1998) mentioned that sclerotic ossicles are absent but scored those bones as present. However, in the holotype of *L. superbus*, Reis (1888, pl. 2. fig. 2) figured 6 small rectangular sclerotic ossicles. In the Bamberg Museum (State of Bavaria, Germany), there is a specimen (NKMB-P-Watt 08/212) identified as an *Undina penicillata* that is in fact a specimen of *Libys* sp. according to the presence of a continuous groove crossed by pillars for the supraorbital sensory canal and many other anatomical characteristics. This undescribed specimen figured by Mäuser (2018, fig. 3.2) shows well preserved sclerotic ossicles. Interestingly, this specimen comes from the Wattendorf Plattenkalk (State of Bavaria, Germany) that is dated of the Upper Kimmeridgian (Fürsich et al., 2007) unlike the holotype of *Libys polypterus* that is Tithonian in age (e.g., Forey, 1998).

### Lower jaw

Most of the bones of the lower jaw are not preserved. Only the left prearticular (Part) is partially visible in mesial view. Its surface is entirely covered with tiny round teeth all of the same size arranged in a regular shagreen. The surface of the teeth appears to be smooth (Fig. 3A5).

Above the prearticular is a fragmented bone that could possibly belong to the left angular visible in mesial view.

A gular plate (Gu) is visible as a partial and poorly preserved imprint.

### Neurocranium, palatoquadrate, parasphenoid and gill arches

Because the right lower jaw is missing, the parasphenoid and the palatoquadrate can be observed.

The basisphenoid (Bsph) is almost covered by the palatoquadrate (Figs. 3 and 5). On the visible anterior portion of the basisphenoid, there are two foramens lying one above the other. The upper foramen, larger than the lower one, corresponds probably to the opening for the superficial ophthalmic nerve and the trochlear nerve (s.oph + IV) while the foramen lying anteroventrally correspond to the foramen for the oculomotor nerve (III). Just above the metapterygoid, a strong antotic process (ant.pr) of the basisphenoid is visible.

Posterior to the metapterygoid are preserved fragments of bone that may correspond to the otic shelf (ot.sh?) of the prootic, from which can be distinguished a long bony portion extending posteriorly that could represent the posterior wing of this bone (Figs. 3 and 5). However, this area is so crushed that it is impossible to discern clearly the different bones of this region.

Only the anterior half of the parasphenoid (Par) can be observed, its posterior half being covered by sheet of indeterminate bone (Figs. 3 and 5). Indeed, it seems that the bone extends posteriorly under the bone interpreted as the basisphenoid. The main visible anterior portion bears small tooth. Due to the mode of preservation, it is hard to assess if the toothed area is restricted to the anterior half or extends more posteriorly. Anteriorly, the parasphenoid expands and is upturned dorsally indicating the presence of a prominent lateral wing (a.w.Par), as for instance in *Macropoma* and *Latimeria* (Forey, 1998). The contact between the parasphenoid and the basisphenoid, and then the contact with the processus connectens, cannot be observed because a sheet of indeterminate bone lies upon this area. It is probable that the buccohypophysial canal is closed, but this characteristic is difficult to assert with accuracy.

The right palatoquadrate is well preserved and consists of a pterygoid (Pt) with a quadrate (Q) posteroventrally and a metapterygoid (Mpt) dorsally (Figs. 3 and 5). The pterygoid is triangular being as long as it is high and similar to the palatoquadrate of *Macropoma lewesiensis* (Forey, 1998, Fig. 7.2) and *Libys polypterus*. The ventral swelling of the pterygoid is well defined as in for instance *Macropoma lewesiensis*, *Libys polypterus* or *Megalocoelacanthus dobiei* (Dutel et al., 2012). The exact shape of the dorsal portion of the metapterygoid is difficult to clearly assess due to its state of preservation.

The palatoquadrate covers the branchial elements (Cb) that can nevertheless be partly observed posteriorly (Figs. 3 and 5). The exact number of these elements is difficult to assess. Some tooth plates (t.p) with tiny granular teeth accompanied by one or two sharp teeth are preserved on some of the branchial elements.

### Axial skeleton

The vertebral column is composed of 44 to 47 neural arches (n.a) and 18 to 20 haemal arches (h.a) (Figs. 3 and 4). At least 38 neural spines can be clearly observed from the tail to the anterior end of the basal plate of the anterior dorsal fin and 3 from this portion to the back of the skull. In this last portion, there is a pack of superimposed and badly preserved arches of which the number is hard to count but should include between 3 to 6 arches. Those arches lying immediately behind the head (Fig. 3) appear to be broader than the posterior neural arches, being then expanded according to Forey’s (1998) criteria. There are 16 neural arches in the caudal region. Neural and haemal arches are well spaced and are not abutting. With a total of 44 to 47 neural arches, the specimen of Teysachaux has less neural arches than in other coelacanths as for instance *Libys polypterus* with 70 neural arches, including 23 haemal arches, and *Macropoma lewesiensis* with 60 neural arches, including 22 haemal arches (Forey, 1998).

There is a maximum of 22 short ossified ribs (ab.rib) counted on the counterpart (NMBE 5034072), meaning that they were at least 11 pairs (Figs. 3 and 4). According to Lambers (1992) there are 17 pairs of ribs in *Libys polypterus* (‘*L. superbus’*). Those short ossified ribs were probably confined to the posterior third of the abdominal area. In many coelacanths, short ribs are confined in the posterior third of the abdominal area but can be also present in the anterior half of the abdominal region as in *Holophagus gulo* (Forey, 1998, fig. 11.8). However, these short ribs should be distinguished from the long ossified abdominal ribs observed in the thoracic area of other actinistians, more generally in mawsoniid coelacanths, as for instance *Diplurus* or *Chinlea* (Cupello et al., 2017; Forey, 1998).

On the part, in the abdominal region behind the pectoral girdle and below the basal plate of the anterior dorsal fin, are large fragments of bone that represent a large ossified lung (oss.lun) (Figs. 3 and 4). An ossified lung is present in other coelacanths as for instance *Macropoma*, *Libys* and *Undina*, among others (Forey, 1998).

### Paired fins

The basal plates and the girdles are all ossified and are well preserved. The fin rays of the paired and posterior unpaired fins are slender than those of the anterior dorsal fin, being then not expanded unlike in some other coelacanth as for instance *Libys polypterus* (Forey, 1998).

#### Pectoral girdle and fins

All bones of the pectoral girdle are present although badly preserved as imprints and fragmented bones on the part and counterpart, respectively (Figs. 3, 4, 5). All those bones are smooth and are devoid of ornamentation.

The cleithrum (Cl) is long and narrow throughout its entire length, and not expanded dorsally. The clavicle (Cla) is small and tapers anteriorly. The extracleithrum (Ecl) is a small and elongated ovoid bone. Dorsal to the cleithrum is a blade-like shaped anocleithrum (Acl). Forey (1998) mentioned in his emended diagnosis of *Libys polypterus* that the anocleithrum is expanded and scored it as forked. However, Lambers (1992, fig. 1 and pl. 1) described and illustrated a sigmoid shaped anocleithrum on a specimen of *Libys polypterus* (‘*L. superbus’*). The overall pectoral girdle of the specimen of Teysachaux does not present any particular anatomical features, except being particularly narrow as in *Libys polypterus* (‘*L. superbus’*) (Lambers, 1992, fig. 1 and pl. 1) and *Undina penicillata* (Forey, 1998).

On the counterpart, the left scapulocoracoid (Scc) is well preserved and extends posteriorly just above the extracleithrum (Fig. 4A1, B). On the part, the right scapulocoracoid has been shifted from its anatomic position (Figs. 3 and 5). The bone has an articulatory tip that accommodated the mesomers of the pectoral fin. It is worth noting that the articulatory tip and the rest of the bone is entirely ossified, which is remarkable. Indeed, in *Latimeria* and probably in most extinct actinistians, the scapulocoracoid is mostly cartilaginous and only the articulatory tip is ossified (Forey, 1998; Mansuit et al., 2019). The pectoral fin is made of about 18–22 rays.

#### Pelvic girdle and fin

The pelvic girdle (P.b) and one pelvic fin (pelv.f) are preserved on the counterpart (Fig. 4). The pelvic girdle lies in the abdominal area at the same level than the basal plate of the first dorsal fin. The pelvic fin is then located well behind the level of anterior dorsal fin as in *Libys* (Lambers, 1992, fig. 1 and pl. 1; Forey, 1998; Fig. 7A). The pelvic bones are narrow and long with an enlarged posterior portion similar to that of *Libys* (Lambers, 1992, fig. 1 and pl. 1; Forey, 1998; Fig. 7A). As in most actinistians, both pelvic bones remain separate. It is difficult to assess clearly the number of fin rays in the pelvic fin because some of them are preserved one above the other, and because some rays appear to have split during the taphonomical process. Therefore, as 33 hemi-rays are present, at least 17 fin rays formed in pelvic fin. In between the fin rays are thin fragments of bones (Fig. 4A6, B) that are interpreted as ossified axial mesomeres (ax.mes). Although their precise shape cannot be observed because they are partially covered by sediments, the outline of the larger one is reminiscent of another ossified axial mesomere well preserved within the anal fin (Fig. 4A5, B). It should be noted that these two pieces of bone could also form a single larger bone rather than two individual bones.

### Unpaired fins

#### Anterior dorsal fin

The basal plate of the anterior dorsal fin (D1.b) is broken into two parts, the anterior half preserved on the counterpart and the posterior half on the part (Figs. 3 and 4). The basal plate is a rectangular bone strengthened in its ventral portion by a ridge running from the anterior border to the center of the posterior half. The basal plate lies completely above the level of the neural spines and has its ventral margin smooth like in most coelacanths. The anterior dorsal fin (d1.f) is composed of 10 fin rays (Fig. 3). The anterior first ray is comparatively shorter than the other rays. The rays are ornamented with small and prominent denticles (Fig. 3A1). The number of fin rays is the same than in *Libys polypterus* (Forey, 1998). However, the rectangular shape of the basal plate is different from the triangular basal plate of *Libys polypterus* (‘*Libys superbus’*) (Forey, 1998; Lambers, 1992, fig. 1 and pl. 1) and *Macropoma lewesiensis* (Forey, 1998 fig. 11.11).

#### Posterior dorsal fin

On the part, the basal plate (D2.b) and rays of the posterior dorsal fin (d2.f) are preserved while there are only some fin rays on the counterpart (Figs. 3 and 4). About 16 fin rays are counted on the part and the counterpart. The rays are devoid of denticles. The basal plate of the posterior dorsal fin is a bone formed by an ovoid plate extending forward by two thin rods. The two rods of the bifurcated anterior portion are of the same length. The main plate bears on its anterodorsal corner a small bony process developing anteriorly, which is present in *Latimeria* and *Laugia* (Millot & Anthony, 1958, pl. LIXa; Forey, 1998, figs. 8.3a and 8.3c) and potentially in *Piveteauia* (Clément, 1999).

#### Anal fin

The anal basal plate (A.b) is preserved in two halves, one on the part and the other on the counterpart (Figs. 3 and 4). The rays of the anal fin (ana.f) are only preserved on the counterpart and comprise about 20 to 23 rays. The anal fin is positioned as a mirror image of the second dorsal fin. The posterior portion of the bone is rectangular and spatula-shaped widening slightly posteriorly. The anterior part is bifurcated in two long processes symmetrically positioned. These processes are shorter than the two processes of the basal plate of the posterior dorsal fin. It is interesting to note that a small axial mesomere (ax.mes) supporting the fin rays can be observed on the counterpart (Fig. 4A5). Axial mesomeres are rarely preserved because the endoskeleton is usually cartilaginous. The Early Triassic *Laugia* is one of the rare cases where those bones are ossified (Forey, 1998).

#### Caudal fin and supplementary lobe

The caudal fin (cau.f) is preserved on the part and present the diphycercal pattern with a lower and upper lobes separated by a supplementary caudal lobe typical of coelacanths (Fig. 3). The caudal fin contains 16 and about 14 radials (Ra) in the upper and lower lobe, respectively. These bones have proximal and distal expanded extremities. In the upper and probably also in the lower lobe, the first anterior radial doesn’t bear any fin ray. There are 15 and 14 to 16 fin rays in the upper and lower lobes, respectively. According to the definition of Forey (1998), the tail is symmetric. In the upper and the lower lobe, respectively, the sixth and fourth anterior most rays bear sharp and prominent denticles while posteriorly the rays are devoid of any denticles (Fig. 3A1).

The supplementary caudal lobe (sup.cau.f.l) is present but the tip is unfortunately missing (Fig. 3). Although incomplete, the supplementary caudal lobe is comparatively long and prominent appearing to develop apart from the caudal fin profile, which is reminiscent of *Libys polypterus* (‘*L. superbus’*) (Lambers, 1992, fig. 1 and pl. 1B-C) and *Undina penicillata* (Forey, 1998; Fig. 7). This feature is considered in the specimen of Teysachaux, as well as in the genus *Libys* in general, as a generic character rather than the indication of a juvenile stage found in some other examples of coelacanths (e.g., Schultze, 1972). Indeed, in the examined specimens of *Libys* with the supplementary caudal lobe kept in the BSM, the basal plate is always fully ossified indicating an adult state. Therefore, these coelacanths have a supplementary caudal lobe developing well apart from the caudal fin unlike other coelacanths that have a supplementary lobe enclosed in the caudal fin such as for instance *Macropomoides* (Forey, 1998) or *Foreyia* (Cavin et al., 2017, fig. 1).

### Scales

**Fig. 6:**
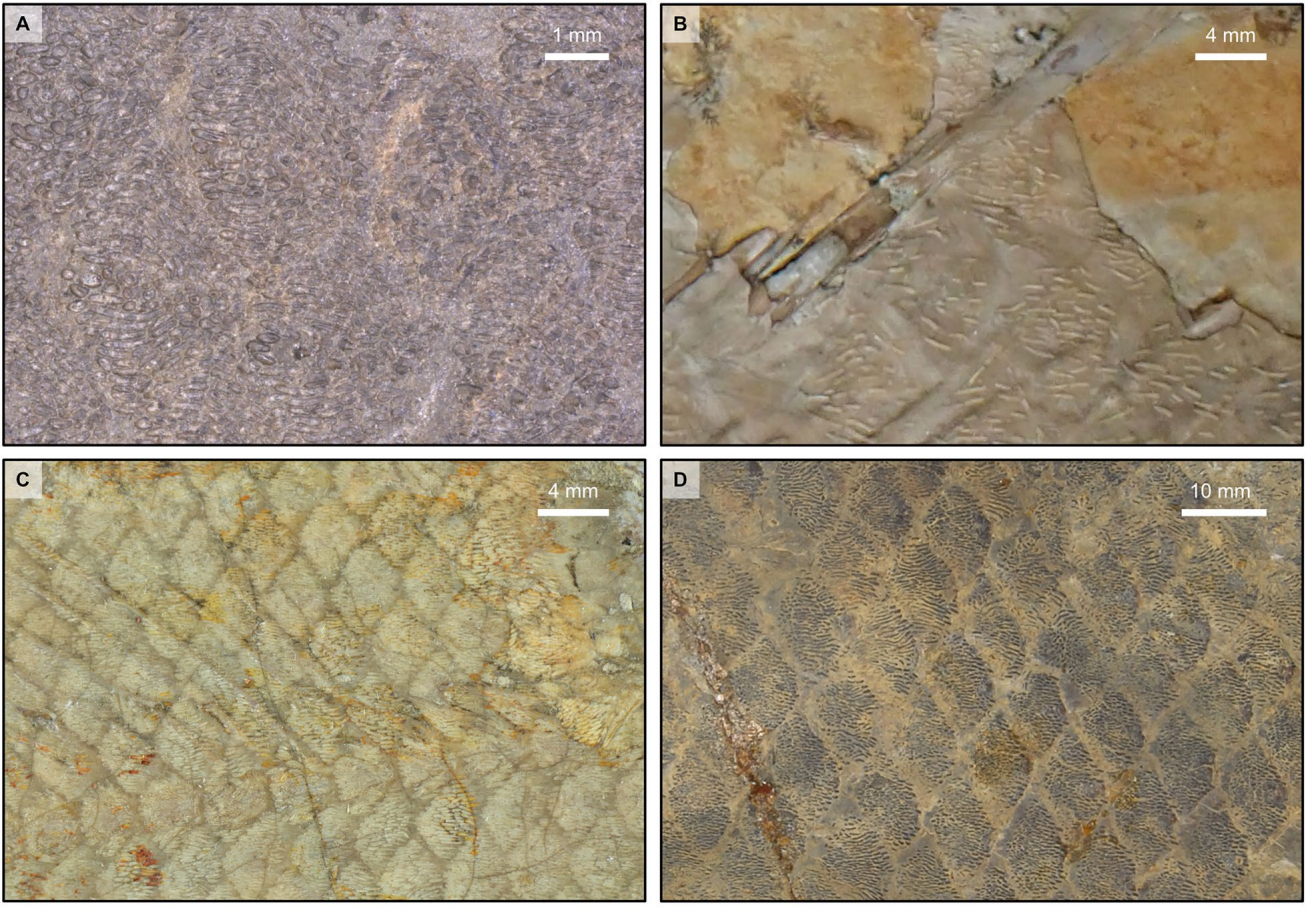
Comparison of scales between *Libys callolepis* sp. nov. and some other Latimeriidae. **A** *Libys callolepis* sp. nov. (holotype, NMBE 5034072). Scales of the flank below the neural arches between the basal plates of the anterior and posterior dorsal fin. **B** *Libys polypterus* (‘*L. superbus’*) (BSM AS I 801a). Scales of the flank located between the neural arches and the basal plate of the second dorsal fin. **C** *Undina penicillata* (BSM 1870 XIV 22). Scales of the middle flank located on the neural arches below the basal plate of the second dorsal fin. **D** *Trachymetopon* (‘*Macropoma’*) *substriolatum* (holotype, SMC J27415) from the Kimmeridgian of Cottenham, Cambridgeshire. Scales of the middle flank. It is worth noting that this species was first attributed by Huxley (1866) to the genus *Macropoma* and latter to *Coccoderma* by Reis (1888). Forey (1998) kept this species within *Coccoderma*, but remarked that it shows affinities with *Holophagus*. Recently, Cavin et al. (2021a) attributed this specimen to the genus of *Trachymetopon* regarding, among other characteristics, the coarse ornamentation on the dermal bones

The scales are well preserved in natural position and exhibit their ornamental pattern, which shows variations according to their position on the body (Fig. 4A2). The exact shape of the entire scales cannot be assessed and only the exposed ornamented part, which is triangular, can be observed. On the flank immediately beneath the first anterior dorsal fin, the scales are strongly ornamented with irregularly sized and elongated round-to-ovoid ridges disposed along a longitudinal axis (Fig. 4A2). Each scale of the lateral line bears three round and pointed tubercles in its middle exposed area that are surrounded by short to elongated ovoid ridges (Fig. 4A3). The pores of the lateral line are very difficult to observe but on some very rare scales, multiple small pores surrounding the central tubercles can be recognized. On the ventral flank, from the pelvic to the anal fins, the scales are ornamented with delicate elongated ridges (Fig. 4A4). No ventral keel scales are present. The patterns of the scales and their distribution on the body of the specimen of Teysachaux are reminiscent of the scales of *Macropoma lewesiensis* as figured by Woodward (1909, pl. 38. fig. 4a–c) and Forey (1998, fig. 11.12a–b). They are different from *Libys polypterus* (Fig. 6B), *Undina penicillata* (Fig. 6C) and *Trachymetopon* (‘*Macropoma’*) *substrialotum* (Fig. 6D), in which the scales are ornamented with dense to sparse ornament of thinner tubercles.

## Discussion

### Identification and comparison of the Teysachaux specimen with other Mesozoic coelacanths

When the specimen was integrated in 1873–1874 within the collection of the Museum of Bern, Fischer-Ooster gave it the name of *‘Macropoma heeri’.* However, Fischer-Ooster never properly published this species with a description and illustration, probably because he passed away in 1875. Since its discovery, the specimen was only cited under the name ‘*Macropoma*’, first by Hug (1898) in his description of the fauna of Les Pueys and, then by Marchant and Pichon (2013), who illustrated it by a photograph in the guide ‘Jurassique Suisse’. Consequently, because the specific name has not been published with an adequate scientific description, and according to the International Code of Zoological Nomenclature rules, the species name ‘*heeri’* is considered as *nomen nudum*.

In 1873–1874, only few genera of coelacanths were known, i.e. *Macropoma*, *Undina*, *Coelacanthus*, *Libys*, *Rhabdoderma*, *Heptanema*, *Coccoderma*, *Holophagus* and *Graphiurichthys*. Thus, it is probable that Fischer-Ooster gave the generic name *Macropoma* because some details, especially the scales, are reminiscent of *Macropoma*. The main question is whether the coelacanth of Teysachaux belongs to a currently known genus and species or represents a new taxon.

**Fig. 7:**
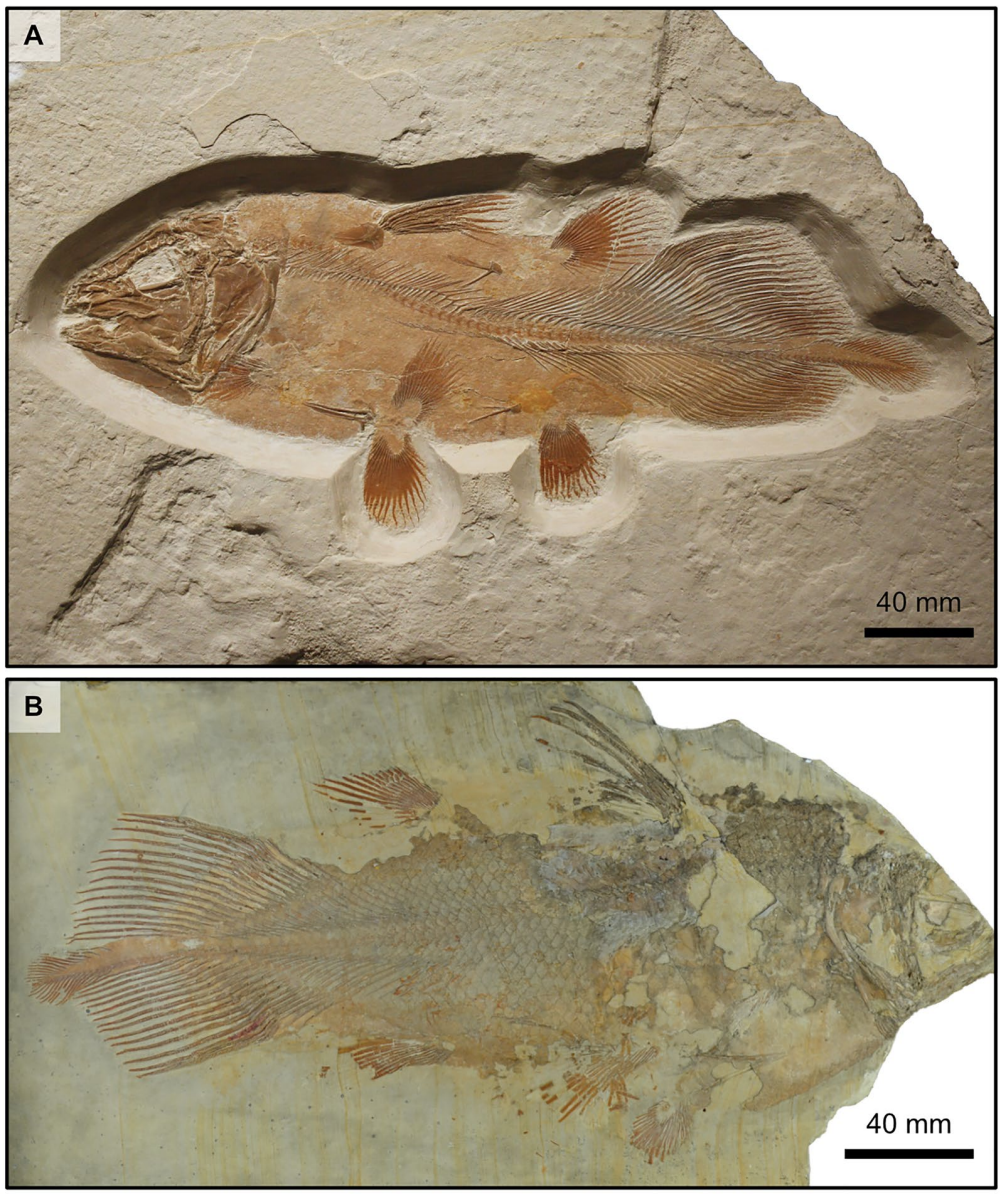
Photographs of two latimeriid coelacanths from the Upper Jurassic of Germany showing the body outline and caudal fin with a prominent supplementary caudal lobe. **A** Specimen of *Libys* sp. (no collection number) exposed in the permanent exhibition of the Bürgermeister-Müller-Museum, Solnhofen, Germany. It is worth noting that this specimen is labeled as *Holophagus*, but the morphological characteristics (e.g., a sensory canal opening through a large groove crossed by pillars) and the meristic clearly indicate that this specimen belongs to the genus *Libys*. **B** *Undina penicillata* (BSM 1870 XIV 22)

The specimen of Teysachaux presents the following combination of characters. One of its most remarkable morphological features is its sensory canal opening though a large groove crossed by pillars. This peculiar characteristic is currently known in *Libys* (Lambers, 1992, fig. 7 pl. 1B-C; Fig. 7A) from the Upper Jurassic of Germany (Forey, 1998) and in *Megalocoelacanthus* from the Upper Cretaceous of the United States (Dutel et al., 2012, figs. 2, 3, 5 and 7). The specimen of Teysachaux is then different from *Macropoma* because in all species of this genus the sensory canals open through many small pores (Forey, 1998).

The consolidated snout of the specimen of Teysachaux is a particular morphological feature found in few coelacanth taxa as *Laugia groenlandica* (Stensiö, 1932, pl. 5. figs. 4–5), *Macropoma lewesiensis* (Forey, 1998, figs. 3.19a and 3.20), *Swenzia latimerae* (Clément, 2005, fig. 4) and *Megalocoelacanthus dobiei* (Dutel et al., 2012, fig. 4). Compared with these taxa, the consolidated snout of the specimen of Teysachaux (Figs. 3A4 and 5) is rather similar to that of *M. lewesiensis* having an anterior opening of the rostral organ occurring as a notch between the ossified snout and the rostral ossicles (Forey, 1998, figs. 3.19a and 3.20a). However, conversely to *M. lewesiensis* the snout of the specimen of Teysachaux is not ornamented. Unfortunately, it is not possible to make a reasonable comparison with the snout of *L. polypterus* because this portion is still imperfectly known and remain to be described on well preserved specimens.

Regarding the meristic features, the specimen of Teysachaux is more closely related to *Libys polypterus* than to *Macropoma lewesiensis*, *Undina penicillata* or *Holophagus gulo* (Table 1). The specimen of Teysachaux and *L. polypterus* have both 10 rays in the anterior dorsal fin, while *H. gulo* has 10–11 rays and *M. lewesiensis* and *U. penicillata* have both less rays with 7 and 8 rays, respectively (Forey, 1998). Although sharing similarities with *L. polypterus*, the specimen of Teysachaux has 44 to 47 neural arches in the vertebral column, which is less than in *M. lewesiensis*, *H. gulo*, *L. polypterus* and *U. penicillata* that have 60, 65, 70 and 70–72 neural arches, respectively (Forey, 1998). Forey (1998) remarked that there is considerable variation among coelacanths regarding the number and the relative spacing of the neural arches. According to Forey (1998), plesiomorphic coelacanths have fewer neural arches than derived taxa. While noting that there are some exceptions, Forey (1998) gave the example of *Rhabdoderma* with 45 neural arches as representing a plesiomorphic state and *Latimeria* with about 110 neural arches. Therefore, compared to *L. polypterus*, *M. lewesiensis*, *H. gulo* and *U. penicillata*, the specimen of Teysachaux represent a more plesiomorphic state.

**Table 1:** Meristic of some Latimeriidae compared to *Libys callolepis* sp. nov. Dataset from Forey (1998)

Taxon/No. of elements	d1.f	d2.f	pect.f	pelv.f	ana.f	cau.f (up.\lo.lobe)	n.a
*Latimeria chalumnae*	8	29–31	30–32	33	29–32	22–25/21–22	93–94
*Macropoma lewesiensis*	7	17	16	18	16	18–20/15–18	60
*Undina penicillata*	8	15–17	19	21	14	18/16	70–72
*Holophagus gulo*	10–11	20	23	18	17	18/17	65
*Libys polypterus*	10	15–20	16	19	18–20	21/19	70
*Libys calloelpis* sp. nov.	10	16	18–22	≥ 17	20–23	15/14–16	44–47

The prominent supplementary caudal lobe of the specimen of Teysachaux is clearly reminiscent of the prominent supplementary caudal lobe of the genus *Libys* (Lambers, 1992, fig. 1, pls 1A-B and 2A; Fig. 7A). The supplementary caudal lobe of *Undina penicillata* (Fig. 7B) is also long (Forey, 1998) but much less prominent than in the specimen of Teysachaux and *L. polypterus*. Unfortunately, the supplementary caudal lobe of *Macropoma* is unknown (Forey, 1998).

The ornamentation of the scales of the specimen of Teysachaux is reminiscent of the ornamentation of the scales of *Macropoma lewesiensis* (Forey, 1998, fig. 11.12 a-b) being then different from *Libys polypterus* (Fig. 6B). Furthermore, the variation of the type of ornamentation according to the position on the body is also similar in the specimen of Teysachaux (Fig. 4A3-4A4) and in *Macropoma lewesiensis* (Forey, 1998, figs. 11.12 a-b).

It is also important to make some comparative remarks with *Undina (?) barroviensis*, which is a poorly defined species of uncertain affinities described first by Woodward (1890) from the Lower Jurassic of England. According to Forey (1998) *Undina (?) barroviensis *has relatively large supraorbital sensory pores. Forey (1998) used the term "very large pores" to describe the openings of the sensory canal of *Libys polypterus*. It therefore remains to demonstrate that this character described by Forey (1998) is the same as in *L. polypterus*. Among other known characters, Forey (1998) also noticed that in *Undina (?) barroviensis* each scale bears few stout tubercles. The current state of knowledge concerning *Undina (?) barroviensis* is insufficient to make a more relevant comparison with the specimen of Teysachaux. However, *Undina (?) barroviensis* and the specimen of Teysachaux clearly belongs to different species because the number of neural arches in the first species is significantly larger than in the second one, i.e. more than 50 based on a comparison with *Holophagus gulo* made by Forey (1998).

According to the previous remarks, the specimen of Teysachaux shares many characters with the genus *Libys*, i.e. (1) head nearly as deep as long; (2) openings of the sensory canals through large grooves crossed by pillars; (3) dermal bones of the skull smooth and unornamented; (4) deep palatoquadrate with a pronounced ventral swelling; (5) very narrow cleithrum, clavicle and extracleithrum; (6) pelvic fins located well behind the level of the anterior dorsal fin and supported by narrow pelvic bones; (7) same number of rays in the anterior and posterior dorsal fins and in the anal fin; (8) prominent supplementary caudal lobe; (9) rays of the anterior dorsal fin and the caudal fin ornamented with many prominent denticles and; (10) presence of an ossified lung.

Nevertheless, the specimen of Teysachaux presents some different morphological characters from *Libys polypterus*, such as: (1) a postparietal shield about half the length of the parietonasal shield (the parietonasal shield is then proportionally shorter than in *L. polypterus*); (2) the teeth covering the prearticular are very small, rounded and smooth; (3) less neural and haemal arches; (4) all fin rays are slender and then not expanded and; (5) scales strongly ornamented with irregularly sized and elongated round-to-ovoid ridges disposed along a longitudinal axis.

Therefore, the specimen of Teysachaux does not belong to the genus *Macropoma* as suggested by von Fischer-Ooster. The specimen of Teysachaux is reminiscent of *Libys polypterus* Münster 1842 but based on morphological differences noted above, it should be regarded as a new species of *Libys*. Therefore, we placed the specimen described here in a new species of the genus *Libys* and erect the new species *L. callolepis* sp. nov.

### Implication for the evolutionary history of coelacanths

In the Tithonian (Upper Jurassic) of the Solnhofen region (Bavaria, Germany), two species of *Libys* have originally been recognized: *L. polypterus* Münster, 1842, and *L. superbus* Zittel, 1887. However, Forey (1998) and subsequent authors considered *L. superbus* to be the junior synonym of *L. polypterus*. The presence of a new species of *Libys* in the Toarcian of Teysachaux (Canton of Fribourg, Switzerland) extends the stratigraphic range of this genus by about 34 million years. This time interval may seem long, but it is comparable to the estimated time interval for the extant *Latimeria* (Inoue et al., 2005; Sudarto et al., 2010) and the stratigraphic range is still longer for 12 extinct genera of coelacanths (Cavin et al., 2021b). In the most recent phylogenies of coelacanths (e.g., Toriño et al., 2021), *Libys* is resolved as the sister genus of the Upper Cretaceous *Megalocoelacanthus*, both branching in the family Latimeriidae. Although the stratigraphic range of the genus *Libys* has expanded considerably, its geographical distribution has not considerably increased. However, the Middle Jurassic fossil record of coelacanths, separating the Lower Jurassic Swiss occurrence of *L. callolepis* sp. nov. from the Late Jurassic German occurrence of *L. polypterus*, is particularly poor with only one other genus recorded.

**Fig. 8:**
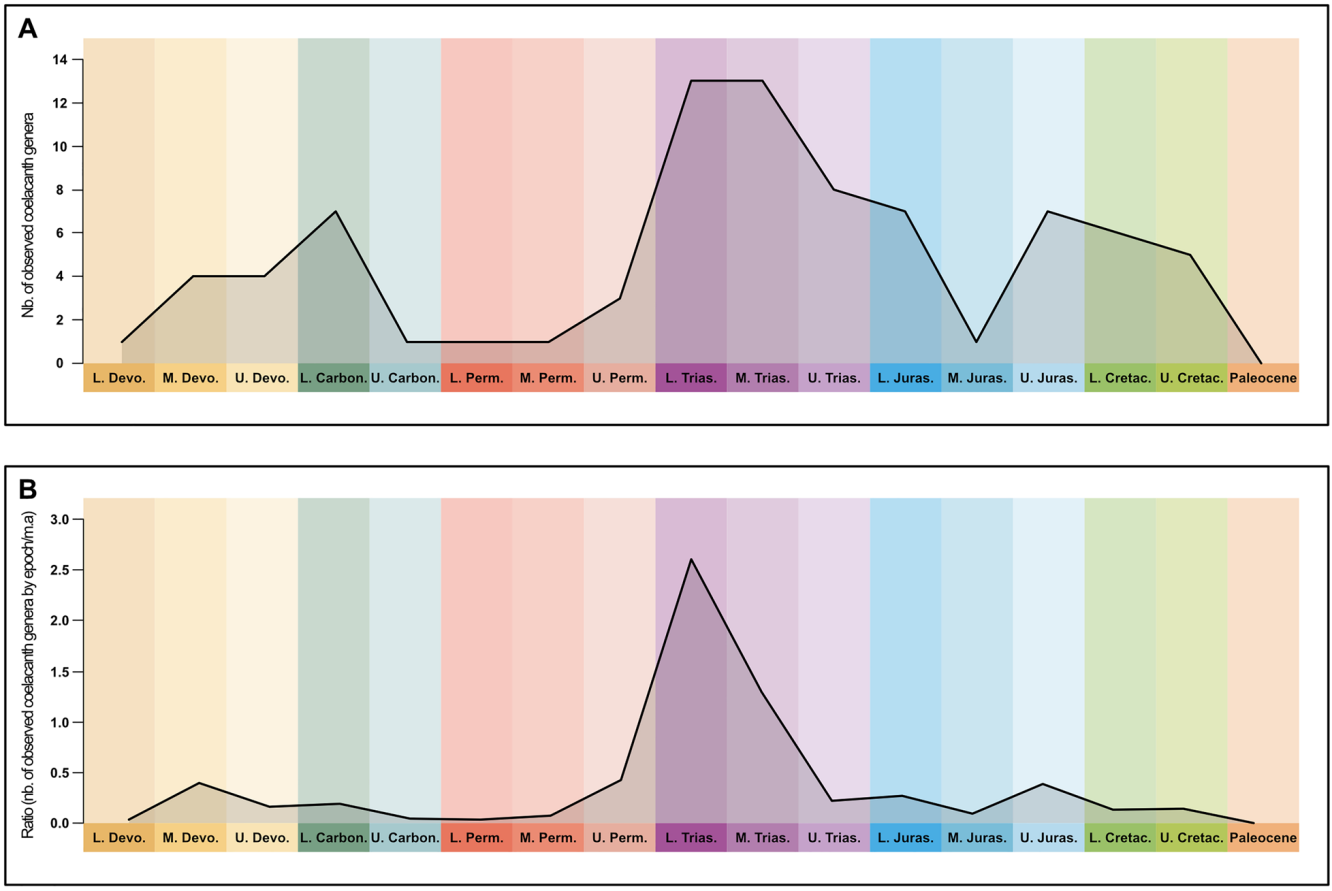
Taxic diversity of coelacanths through time. **A** Number of observed coelacanth genera by Epoch and **B** Ratio of coelacanth genera by Epoch, calculated as the number of genera by Epoch per million of years. Dataset after Cavin et al. (2021a), with the addition of the occurrence of *Libys* in the Lower Jurassic

The taxic diversity of the coelacanths estimated on the basis of the fossil record has always been low since the Lower Devonian. The diversity shows some vagaries over time with periods of proportionally high and low diversity (Fig. 8a). However, when calculated per million years rather than raw diversity, the peak and the falls are smoothed (Fig. 8b). In this pattern, coelacanths experienced a very high peak in taxa diversity after the Permo-Triassic mass extinction in the Lower Triassic and to a lesser degree in the Middle Triassic. From the beginning of the Upper Triassic, the taxic diversity of the group shows a significant steady decrease until the Middle Jurassic where it reaches its minimum with a single known species. The cause of the decline of coelacanths may be related to competition with neopterygians and chondrichthyans due to their slow metabolism. Indeed, compared to other bony fish groups, Neopterygians show a continuous increase in diversity from the Middle-Upper Triassic (Romano et al., 2016).

Alongside a peak of diversity of coelacanths (e.g., Cavin et al., 2013), the Early and Middle Triassic also witnessed a high morphological disparity, exemplified by the strange *Foreyia* from the Prosanto Formation (Canton of Graubünden) (Cavin et al., 2017), and by another morphologically aberrant taxon from the Besano Formation (Canton of Ticino), under study (Ferrante et al., 2017; Ferrante et al., work in progress). The appearance of *Libys callolepis* sp. nov. from the Lower Jurassic announces the beginning of a new stage in the evolution of the coelacanths, with the appearance of taxa with morphological Bauplan which does not derogate from the general coelacanth Bauplan (except in disparity of body size (Cavin et al., 2021a)) and with long stratigraphic ranges and/or ghost ranges (Toriño et al., 2021).

## Conclusion

*Libys callolepis* sp. nov. represents the only Swiss occurrence of a coelacanth, with the exception of those from the rich Middle Triassic sites of the Prosanto Formation and of Monte San Giorgio. Its presence in the Lower Jurassic beds extends the stratigraphic range of the genus *Libys* by about 34 million years, but without increasing considerably its geographic distribution. The genus *Libys* belongs to the modern family Latimeriidae and the appearance of *L. callolepis* sp. nov. heralds a long period, up to the present day, of coelacanth genera with very long stratigraphic range and reduced morphological disparity, which have earned them the nickname of ‘living fossils’.

## Data Availability

The holotype (NMBE 5034072 and 5034073) is kept in the collections of the Natural History Museum Bern (Canton of Bern, Switzerland). The dataset used to perform the taxic diversity of coelacanths through time can be found as a supplementary information from the paper of “Cavin, L., Piuz, A., Ferrante, C., & Guinot, G. (2021a). Giant Mesozoic coelacanths (Osteichthyes, Actinistia) reveal high body size disparity decoupled from taxic diversity. Scientific reports, 11(1), 1–13. https://doi.org/10.1038/s41598-021-90962-5”.

## Abbreviations

NMBE: Naturhistorisches Museum Bern, Bern, Switzerland
BSM: Bayerische Staatssammlung für Paläontologie und Geologie, Munich, Germany
SMC: Sedgwick Museum, Cambridge University, England
A.b: Basal plate of the anal fin
a.ros: Anterior opening for the rostral organ
a.w.Par: Ascending wing of parasphenoid
ab.rib: Abdominal rib
ax.mes: Axial mesomere
Acl: Anocleithrum
ana.f: Anal fin
ant.pr: Antotic process
Bsph: Basisphenoid
cau.f: Caudal fin
Cb: Ceratobranchial
ch.bo: Cheek bone
Cl: Cleithrum
Cla: Clavicle
D1.b: Basal plate of the anterior dorsal fin
D2.b: Basal plate of the posterior dorsal fin
d1.f: Anterior dorsal fin
d2.f: Posterior dorsal fin
Ecl: Extracleithrum
Ext.l: Lateral extrascapular
Gu: Gular plate
h.a: Haemal arches
L.r: Lateral rostral
Mpt: Metapterygoid
n.a: Neural arches
Na: Nasal
Op: Opercle
oss.lun: Ossified lung
Ot.sh: Otic shelf
ot.s.c: Otic sensory canal
Pa: Parietal
Par: Parasphenoid
Part: Prearticular
P.b: Pelvic bone
pect.f: Pectoral fin
pelv.f: Pelvic fin
Pp: Postparietal
Pt: Pterygoid
Q: Quadrate
Ra: Radial
Ros.Pmx: Rostropremaxilla
s.oph + IV: Opening for the superficial ophthalmic nerve and the trochlear nerve
Scc: Scapulocoracoid
So: Supraorbital
so.s.c: Supraorbital sensory canal
Stt: Supratemporal
sup.cau.f.l: Supplementary caudal fin lobe
t.p: Tooth plate
Te: Tectal
v.pr.Pp: Ventral descending process of the postparietal
v.pr.stt: Ventral descending process of supratemporal
III: Foramen for the oculomotor nerve
